# Whole Genome Sequencing of Hulunbuir Short-Tailed Sheep for Identifying Candidate Genes Related to the Short-Tail Phenotype

**DOI:** 10.1534/g3.117.300307

**Published:** 2017-12-05

**Authors:** Dafu Zhi, Lai Da, Moning Liu, Chen Cheng, Yukun Zhang, Xin Wang, Xiunan Li, Zhipeng Tian, Yanyan Yang, Tingyi He, Xin Long, Wei Wei, Guifang Cao

**Affiliations:** * College of Veterinary Medicine, Inner Mongolia Agricultural University, Huhhot 010018, People’s Republic of China; † Inner Mongolia Academy of Agricultural and Animal Husbandry Sciences, Huhhot 010018, People’s Republic of China; ‡ College of Life Sciences, Inner Mongolia Agricultural University, Huhhot 010018, People’s Republic of China

**Keywords:** Hulunbuir short-tailed sheep, short-tail phenotype, whole genome sequencing, selective sweep, *T/*Brachyury gene, Genome Report

## Abstract

The Hulunbuir short-tailed sheep (*Ovis aries*) is a breed native to China, in which the short-tail phenotype is the result of artificial and natural selection favoring a specific set of genetic mutations. Here, we analyzed the genetic differences between short-tail and normal-tail phenotypes at the genomic level. Selection signals were identified in genome-wide sequences. From 16 sheep, we identified 72,101,346 single nucleotide polymorphisms. Selection signals were detected based on the fixation index and heterozygosity. Seven genomic regions under putative selection were identified, and these regions contained nine genes. Among these genes, *T* was the strongest candidate as *T* is related to vertebral development. In *T*, a nonsynonymous mutation at c.G334T resulted in p.G112W substitution. We inferred that the c.G334T mutation in *T* leads to functional changes in Brachyury—encoded by this gene—resulting in the short-tail phenotype. Our findings provide a valuable insight into the development of the short-tail phenotype in sheep and other short-tailed animals.

Sheep were among the earliest domesticated herbivores. Sheep domestication dates back to the end of the Mesolithic period, ∼11,000 yr ago (Chessa *et al.* 2009). Domestication and artificial selection have led to marked changes in sheep behavior, appearance, and other important traits (Megens *et al.* 2008). Most mammal tails are used for balance, communication, and attack. However, in sheep, tails store energy. The fat-tail phenotype is a trait necessary for survival in harsh environments (Pourlis 2011). It is exhibited by Hulunbuir short-tailed sheep, which have been bred for the past century by local herdsmen of the Hulunbuir grassland—a world-renowned highland pasture in arid and semi-arid regions of North China characterized by a short frost-free period, long and cold winter, and dry season constituting a considerable part of the year (Yang *et al.* 2012). The tails of Hulunbuir short-tailed sheep (Figure 1A) are of variable length, and are generally classified into two categories: extremely short, a tail exposing the anus (Figure 1C); and moderately short, a tail covering the anus (Figure 1D).

**Figure 1:**
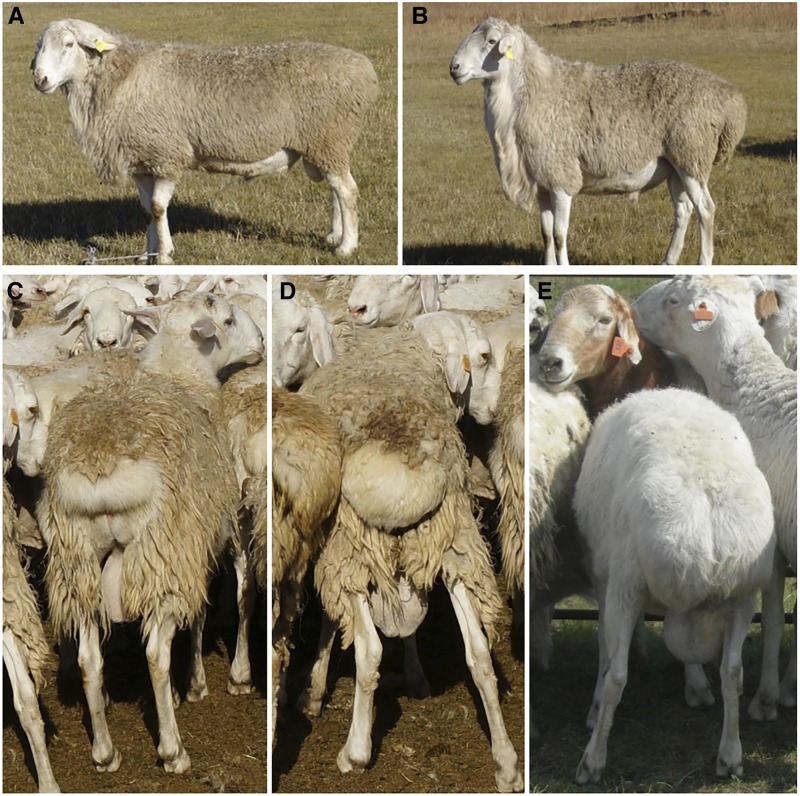
Tail phenotypes of sheep. (A) Hulunbuir short-tailed sheep; (B) Barag sheep; (C) extremely short in Hulunbuir short-tailed sheep; (D) moderately short in Hulunbuir short-tailed sheep; (E) normal tail in Barag sheep.

Several candidate genes—including *HES7* (Bessho *et al.* 2001), *PAX1* (Wilm *et al.* 1998), *T* (Smith 1999), and *WNT3A* (Greco *et al.* 1996)—related to vertebral development in laboratory mice are reported to be related to the short-tail phenotype; however, the determinant genes related to the short-tail phenotype in sheep remain to be identified.

Recently, many genomic regions under putative selection—which may be related to domestication, adaptation, and other important traits—have been reported in chickens, cats, dogs, and pigs (Rubin *et al.* 2010; Axelsson *et al.* 2013; Moon *et al.* 2015; Xiao *et al.* 2016). However, detection of selection signals within a single species or population cannot determine whether the genomic region is under putative selection or related to genetic drift. Genomic regions under putative selection are identified using the fixation index (F_st_), which is based on significant differences in allele frequencies between two populations. However, F_st_ does not identify the population wherein selection occurs; hence, it cannot be used to determine the direction of selection. Therefore, in the present study, we analyzed heterozygosity (H_p_) to identify the specific genomic regions under putative selection. Axelsson *et al.* (2013) combined F_st_ and H_p_ to locate the genomic regions under putative selection, and the corresponding genes during dog domestication. Here, we applied the methodology of Axelsson *et al.* (2013) to locate the genomic regions related to the short-tail phenotype in Hulunbuir short-tailed sheep.

To verify the role of the determinant genes and identify novel genes regulating the short-tail phenotype, we selected sheep with extremely short tails from a population of Hulunbuir short-tailed sheep, and performed whole genome sequencing. The short-tail phenotype has previously been reported in Australian Merino sheep (James *et al.* 1991); however, to the best of our knowledge, we are the first to conduct molecular studies of the short-tail phenotype in sheep.

## Materials and Methods

### Ethics statement

All animal care and experiments were conducted in accordance with the *Guide for the Care and Use of Laboratory Animals*, and were approved by the Institutional Animal Care and Use Committee of Inner Mongolia Agricultural University.

### Samples

Hulunbuir short-tailed sheep and Barag sheep were selected from the Autonomous County of Evenki in the Inner Mongolia Autonomous Region, China. Barag sheep (Figure 1B), which resemble Hulunbuir short-tailed sheep but have a normal tail morphology (Figure 1E), served as controls. We randomly selected 100 short-tailed sheep and 100 Barag sheep for tail-length measurement. Caudal vertebrae were collected from 10 short-tailed sheep and 10 Barag sheep. Blood samples were collected from the following sheep—eight unrelated 2-yr-old short-tailed sheep (four males and four females) with extremely short tails, and eight unrelated 2-yr-old Barag sheep (four males and four females) with normal tails were selected for whole genome sequencing; 120 short-tailed sheep and 110 Barag sheep were randomly selected to confirm the mutation site; ∼2 ml of blood was collected in EDTA-containing vacutainers and stored in liquid nitrogen (−196°). Genomic DNA was extracted from the whole blood samples using the DNeasy Blood and Tissue Kit (Qiagen, Duesseldorf, Germany).

### X-ray analysis and specimen preparation

X-ray images of the caudal vertebrae were captured from the front using the ClearVet DR16 Imaging System. Deposits and muscle were carefully peeled off from the caudal vertebrae. The caudal vertebrae were fixed in ethanol for 5 d, and cleared by immersing in 0.5% NaOH for 2 d. To remove fat, oil, and adipose tissue, the caudal vertebrae were immersed in petrol for 3 d. Data were recorded and saved according to the order of X-ray images.

### Sequencing and SNP calling

A sequencing library with an average insert size of 350 bp was constructed for each sample. Sequencing was performed on the Illumina Hisequation 2000 sequencer at Novogene Corporation to generate 150-bp paired-end reads; ∼13.1 Gb of high-quality data were generated, achieving an average fivefold genome coverage for each individual. The clean data were aligned to the sheep reference genome (Oar_v3.1 http://asia.ensembl.org/Ovis_aries/Info/Index) using Burrows-Wheeler Aligner 0.6.1 with parameters set as “mem -t 4 -k 32 -M” (Li and Durbin 2010); duplications were eliminated from the alignments using SAMtools with parameters set as “rmdup.” Single nucleotide polymorphism (SNPs) were detected using SAMtools with parameters set as “mpileup-m 2 -F 0.002 -d 1000” and annotated using ANNOVAR (Li *et al.* 2009; Wang *et al.* 2010). SNPs with low-quality scores (GQ < 20) and inter-SNP distance of <5 bp were filtered.

### Selective sweep analysis

A sliding-window approach (100-kb windows sliding in 10-kb steps) was employed for quantifying H_p_ in the short-tailed sheep and F_st_ between the short-tailed and Barag sheep (Li *et al.* 2014). H_p_ was calculated using the formula $H_{p}=2\sum^{\;}n\mathrm{Maj}\;\sum^{\;}n\mathrm{Min}/{\left(\sum^{\;}n\mathrm{Maj}+\sum^{\;}n\mathrm{Min}\right)}^{2},$ where $\sum^{\;}n\text{Maj}\;$and $\sum^{\text{​}}n\mathrm{Min}$ are the sum of nMaj and nMin, respectively. We transformed the H_p_ values into Z-scores using the formula $ZH_{p}=\left(H_{p}-\mu H_{p}\right)/\sigma H_{p},$ where $\mu H_{p}$ is the overall average heterozygosity and$\sigma H_{p}$ is the SD for all windows within the group. Genetic differentiation between the short-tailed and Barag sheep was measured using fixation index (F_st_) with the formula $F_{st\;}=1-p_{1}q_{1}+p_{2}q_{2}/2p_{r}q_{r},$ where $p_{1},$$p_{2}$ and $q_{1},$$q_{2}$ represent the frequencies of alleles A and a in populations of Hulunbuir short-tailed sheep and Barag sheep, respectively, and $p_{r}$ and $q_{r}$ represent the frequencies of alleles A and a, respectively, in the whole population (Clark and Hartl 2007). F_st_ values were Z-transformed using the formula for ZH_p_. The H_p_ and F_st_ data were calculated based on the R language package (http://www.r-project.org/). The selected regions were defined as genetic regions in overlapping windows with extremely low ZH_p_ values (ZH_p_ < −5) and extremely high ZF_st_ values (ZF_st_ > 4.5).

### T sequencing

The primer pairs for the amplification of *T* exons were designed based on the sheep genome assembly (Oar_v3.1). The primer sequences are provided in Supplemental Material, Table S1. Sanger sequences of PCR duplicates were detected by Invitrogen Corporation, Shanghai, China.

### Data availability

The Illumina sequence reads were deposited in the NCBI Sequence Read Archive under the accession number SRP106953. The authors state that all data necessary for confirming the conclusions presented in the article are represented fully within the article.

## Results

### Measurements

**Figure 2:**
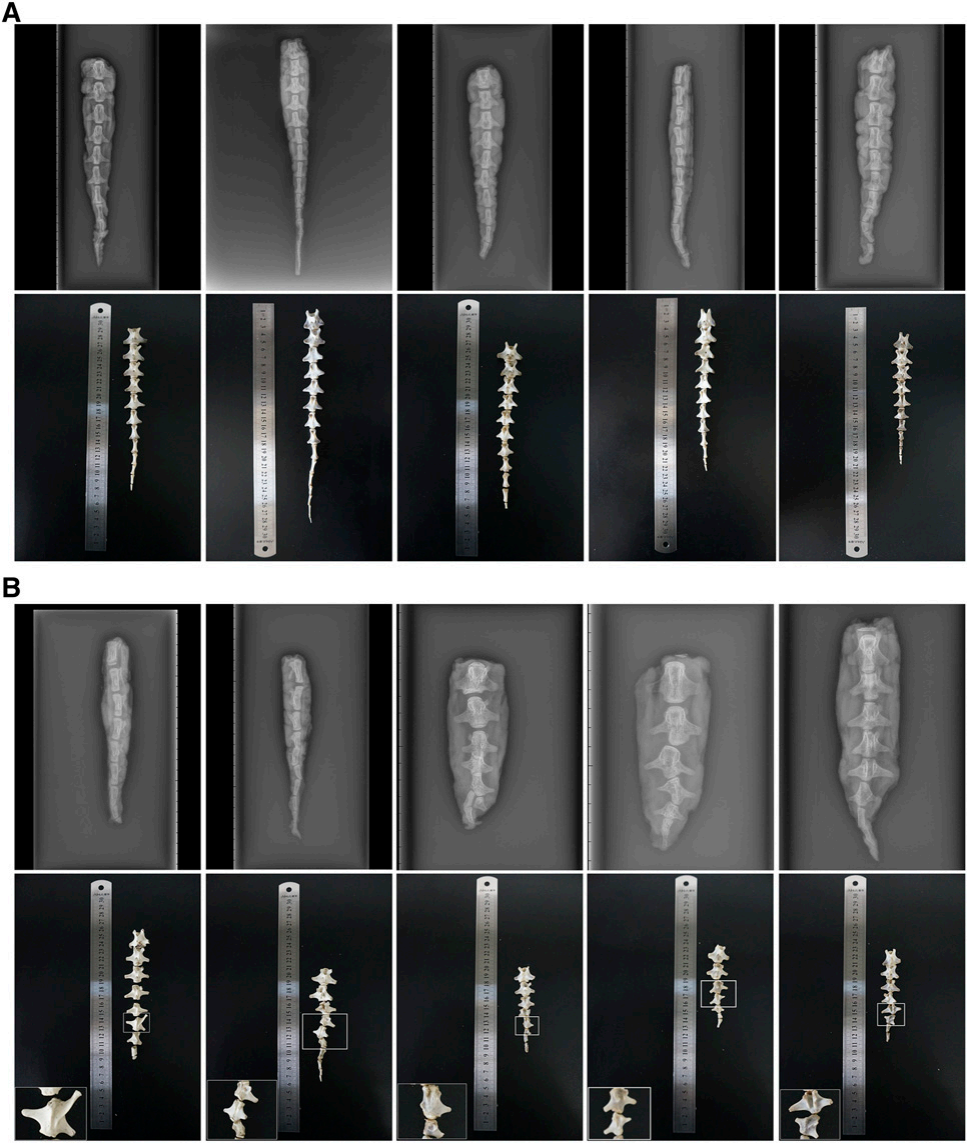
Phenotype of caudal vertebrae in the two populations. (A) Phenotype of the caudal vertebrae in Barag sheep; (B) phenotype of the caudal vertebrae in Hulunbuir short-tailed sheep. Top rows show X-rays and bottom rows show the caudal vertebrae that were placed based on the X-rays in (A and B).

The average tail lengths of the Barag and Hulunbuir short-tailed sheep were 26.32 and 16.01 cm, respectively. The average lengths of the caudal vertebrae in the Barag and Hulunbuir short-tailed sheep were 21.12 and 13.86 cm, respectively. Deformed caudal vertebrae were observed in seven Hulunbuir short-tailed sheep (Figure 2).

### Genome resequencing

Eight sheep with extremely short tails were selected from the short-tailed sheep, and eight Barag sheep with normal tails were selected. We performed genome sequencing for the 16 sheep and obtained 232.23 Gb of paired-end DNA reads. Of this, 231.07 Gb (99.50%) consisted of high-quality paired-end reads (Q20 ≥ 94.33% and Q30 ≥ 88.47%) that could be aligned to the sheep reference genome (Oar_v3.1) (Table S2). For individual sheep, the alignment rate was 98.13–98.75%. For the reference genome (excluding N’s), the average coverage depth was 4.59–5.27×. The reads with 1× coverage depth (covered by at least one base) accounted for >94.19% (Table S3). The read alignments were normal, and the reads were eligible for variation detection and other analyses.

### SNP and selective sweep

**Figure 3:**
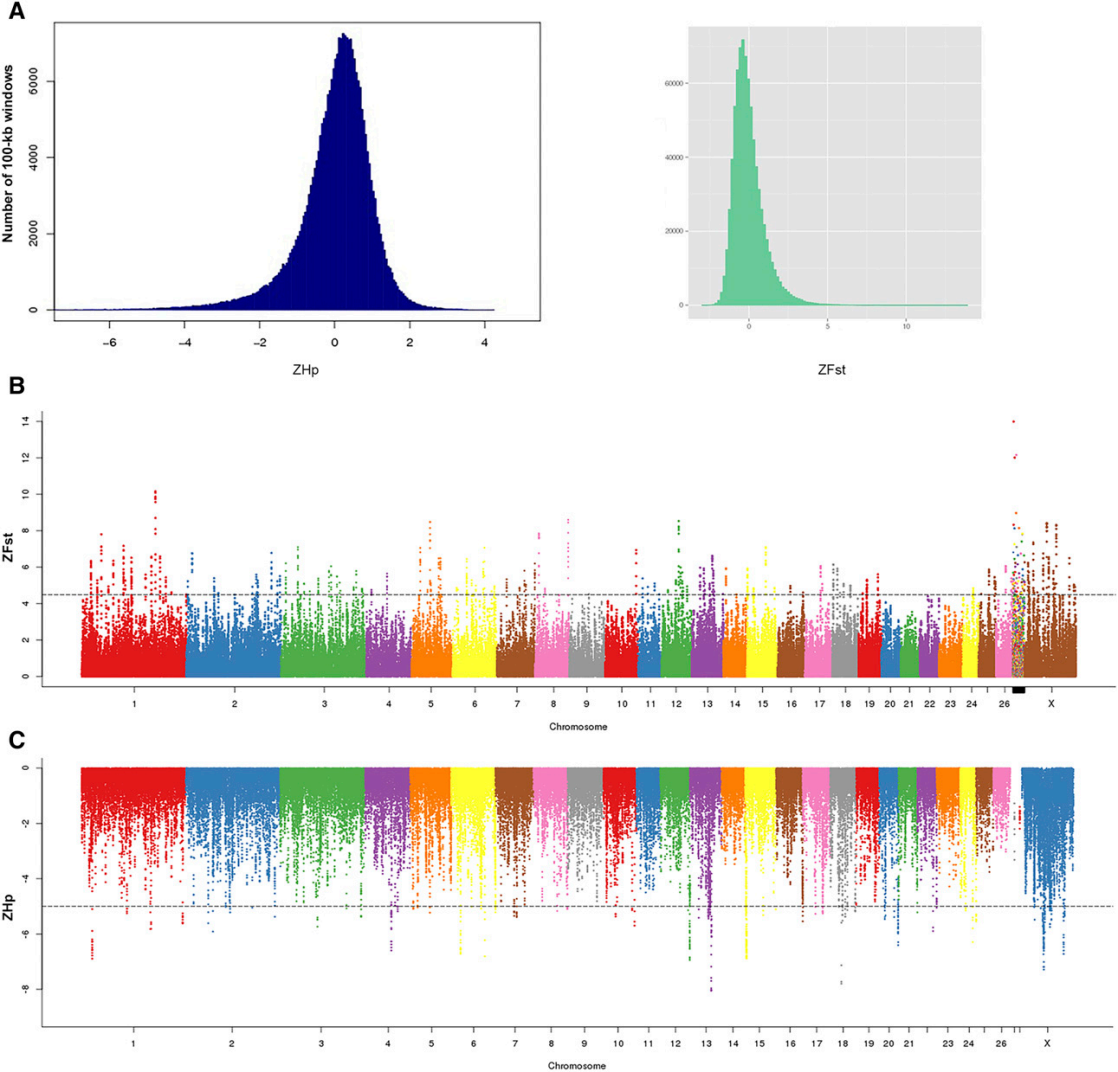
Selective analysis of the sheep genome. (A) Distribution of Z-transformed average heterozygosity in the Hulunbuir short-tailed sheep and Z-transformed average fixation index (ZF_st_) for autosomal 100-kb windows. (B) Positive end of the (ZF_st_) distribution plotted along chromosomes (chromosomes are separated by color); horizontal dashed line indicates the cut-off (ZF_st_ > 4.5) used for extracting outliers. (C) Negative end of the (ZH_p_) distribution plotted along chromosomes (chromosomes are separated by color); horizontal dashed line indicates the cut-off (ZH_p_ < −5) used for extracting outliers.

In the 16 sheep, we identified 72,101,346 SNPs, of which 492,307 SNPs were localized to coding regions, and responsible for 205,408 nonsynonymous nucleotide substitutions (202,338 missense, 2905 stop-gains, and 165 stop-losses) (Table S4). According to the SNP densities in the genome and reference genome assembly, the number of SNPs in the sliding window was <20 beginning from 100 kb; this trend persisted. Additionally, the regions containing <20 SNPs were stable. Therefore, the width of the sliding window was selected as 100 kb. Alignments were performed between the two populations, using a 50%-overlapping interval as the step length. For each window, F_st_ was calculated and Z-transformed into a ZF_st_ value that obeyed standard normal distribution (Figure 3A). The larger the ZF_st_ value, the greater the genetic differentiation between the populations. H_p_ was evaluated for the short-tailed sheep population with the same sliding-window parameters, and it was Z-transformed to standard normal distribution for the population (Figure 3A). Based on the Z value, we observed considerable genetic differentiation in many genomic regions between the populations, including those unrelated to tail morphology. To determine whether certain genetically different genomic regions were related to tail morphology, we overlapped the small interval of ZH_p_ and large interval of ZF_st_. We set ZF_st_ = 4.5 as the threshold for identifying the genomic regions under putative selection and selected windows with ZF_st_ values greater than this threshold (Figure 3B). Moreover, we set ZH_p_ = −5 as the threshold and selected windows with ZH_p_ values less than this threshold (Figure 3C). The overlapping regions were identified and combined into seven candidate genomic regions (20–160 kb). These candidate regions covered a genomic length of 790 kb and contained nine genes (Table 1).

**Table 1:** Overlapping genes identified using ZH_p_ and ZF_st_

Chr	Interval Location	ZF_st_	ZH_p_	Candidate Gene	Annotation	Gene Location
1	69800001–69950000	4.9462	−5.4862	*FNBP1L*	Formin-binding protein 1-like	69805549–69915406
1	69800001–69950000	4.7863	−5.9525	*BCAR3*	Breast cancer anti-estrogen resistance 3	69930328–70015474
6	37440001–37580000	4.9422	−5.0057	*LCORL*	Ligand-dependent nuclear receptor corepressor-like	37365236–37452332
8	87770001–87890000	8.4551	−5.1642	*T*	T, Brachybury homolog	87796143–87805552
15	16940001–17060000	4.7804	−6.5384	*SLC35F2*	Solute carrier family 35, member F2	16854039–16994650
15	16940001–17060000	4.6566	−5.9519	*RAB39A*	RAB39A, member RAS oncogene family	166995339–17027728
15	17090001–17100000	4.7602	−6.0915	*CUL5*	Cullin 5	17088942–17210813
X	77440001–77470000	4.8223	−5.1215	*IRAK1*	Interleukin-1 receptor-associated kinase 1	77447119–77450460
X	77440001–77470000	4.8801	−5.0186	*MECP2*	Methyl-CpG-binding protein 2	77461651–77467940

**Figure 4:**
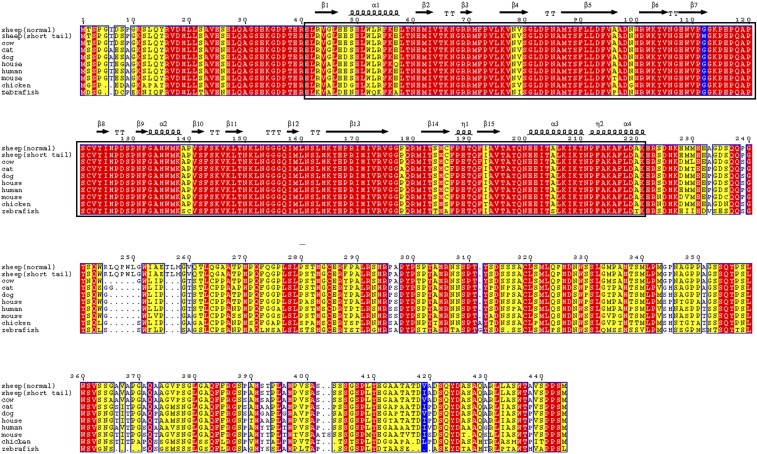
Alignment of amino acid sequences of Brachyury among animals. Brachyury is a protein encoded by *T*. Amino acid residues where p.G112W and p.V419I were located are indicated in blue.​ Identical amino acids, conserved substitutions, and semiconserved substitutions are indicated in red, yellow, and white respectively. Dots represent gaps in the alignment.

Of these nine annotated genes, *T*, which is a gene related to development of the spine in mice (Beddington *et al.* 1992), harbored 10 mutations, including eight synonymous and two nonsynonymous mutations. The two nonsynonymous substitutions in *T* were considered putative mutations, including G-to-T transversion in exon two (c.G334T) and G-to-A transition in exon nine (c.G1255A), which corresponded to glycine-to-tryptophan substitution at amino acid residue 112 (p.G112W) and valine-to-isoleucine substitution at amino acid residue 419 (p.V419I), respectively (Figure 4).

### Validation using Sanger sequencing

The identified nonsynonymous nucleotide substitutions in *T* were verified using Sanger sequencing with a large sample size in 120 short-tailed and 110 Barag sheep. Variation data were deposited in GenBank under the accession number MF996360. We detected the heterozygous form of c.G334T in the short-tailed sheep, but not in the Barag sheep. However, we detected c.G1255A in both populations (Table 2). We inferred that the c.G334T mutation in *T* is the primary cause of the short-tail phenotype. However, this does not exclude the possibility that other genes control the short-tail phenotype.

**Table 2:** Summary of genotypic data in the two populations

Breed		c.G334T			c.G1255A		
	N	(G/G)	(G/T)	(T/T)	(G/G)	(G/A)	(A/A)
Hulunbuir short-tailed sheep	120	17	103	0	81	0	39
Barag sheep	110	110	0	0	97	0	13

## Discussion

Hulunbuir short-tailed sheep are fat-tailed sheep, with large quantities of adipose deposited in their tail regions. Most previous research studies on sheep tails have focused on adipose deposition (Moradi *et al.* 2012; Wang *et al.* 2014; Yuan *et al.* 2017). Liu *et al.* (2015) suggested that tail length in short-tailed sheep is related to adipose deposition. However, we believe that tail length is related to the length of the caudal vertebrae. This prediction was supported by our observation of deformed vertebrae in the tails of seven Hulunbuir short-tailed sheep. The specific molecular mechanism functioning in the short-tail phenotype remains to be elucidated; however, we believe that the mutated *T* gene plays an important role in regulating this phenotype. Of the nine investigated genes, *BCAR3* (Agthoven *et al.* 1998), *FNBP1L* (Huett *et al.* 2009), *IRAK1* (Li *et al.* 2015), *CUL5* (Byrd *et al.* 1997), and *RAB39A* (Seto *et al.* 2013) are related to the immune system; *MECP2* (Tao *et al.* 2009) and *SLC35F2* (Sonuga-Barke 2013) are related to neurodevelopment; and *LCORL* (Signer-Hasler *et al.* 2012) is related to skeletal frame size. Only the *T* gene is related to development of the spine, and this gene seems to be associated with a short-tail phenotype in mice (Beddington *et al.* 1992).

We identified seven candidate genomic regions that included *T*. This gene was further verified using Sanger sequencing. *T* encodes a transcription factor named Brachyury, which is the key regulator of mesoderm formation during early development. The Brachyury protein is an important functional transcription factor, in which ∼180 amino acid residues located near the N-terminus display DNA-binding activity; this is the T domain. This region can specifically bind to a 5-bp functional domain in DNA (TCACA) (Kispert and Herrmann 1993; Palena *et al.* 2007; Fernando *et al.* 2010). Brachyury is specifically expressed in the notochord of the mesoderm during gastrulation, and it regulates growth and development of the embryonic notochord. However, Brachyury is not expressed during mid-to-late pregnancy (Sangoi *et al.* 2011).

As early as 1927, Dobrovolskaïa-Zavadskaïa described the phenotype of a Brachyury mutation in mice (Kavka and Green 1997). Mice heterozygous for the Brachyury mutation had short and slightly curved tails. Homozygous or compound heterozygous mice died *in utero* after ∼10 d of the embryonic period because of failure to form the dorsal cord and allantois (Showell *et al.* 2004). The short-tail phenotype in mice was first discovered in 1927, but it was not until 1990 that the *T* gene was cloned (Herrmann *et al.* 1990). Pennimpede *et al.* (2012) characterized an inducible miRNA-based on an *in vivo* knockdown mouse model of *T*, which exhibited skeletal defects.

We identified two loci of nonsynonymous mutations in *T* using genome sequencing in short-tailed sheep, and we localized the c.G334T mutation in the T domain of the Brachyury gene. Specific regions in the T domain are highly sensitive to structural changes caused by mutations, and these mutations may result in conformational changes in the protein. By combining X-ray diffraction and analysis of the DNA-binding domain in *Xenopus*, Muller observed high similarity between amino acids in this region and those in contact with DNA (Müller and Herrmann 1997). In the present study, we inferred that the p.G112W mutation affects the binding ability of the T domain to DNA.

We did not detect the c.T334T mutation in the short-tailed sheep population, consistent with the theory that homozygotes or compound heterozygotes result in embryonic lethality (Buckingham *et al.* 2013). The c.G334G mutation was detected in individuals with normal tails. Wu *et al.* (2010) observed that, when mice with moderately long tails were mated with short-tailed mice, the offspring showed short-tail and normal-tail phenotypes; however, the genotypes of the offspring were not analyzed. This may explain the phenomenon of the c.G334G mutation in individuals with normal tails in the short-tailed sheep population. Herdsmen continue to breed this group and eliminate individuals with nonideal tail types and other individuals exhibiting poor phenotypes; we propose that this explains the nonconformity of genotypic frequencies with genetic theory.

In conclusion, the results of our present study suggest that the c.G334T mutation in *T* directly results in the short-tail phenotype in sheep. The candidate genes identified in our study provide the basis for understanding the molecular mechanism of the short-tail phenotype in sheep and other short-tailed animals.

## Supplementary Material

Supplemental material is available online at www.g3journal.org/lookup/suppl/doi:10.1534/g3.117.300307/-/DC1.
